# Adhesive capsulitis and dynamic splinting: a controlled, cohort study

**DOI:** 10.1186/1471-2474-10-111

**Published:** 2009-09-07

**Authors:** Paul D Gaspar, F Buck Willis

**Affiliations:** 1 Gaspar Doctors of Physical Therapy, 981 Lomas Santa Fe, Ste. A Solana Beach, CA 92075, USA; 2 Texas State University, HPER Adjunct Faculty (at the time of this study), Dynasplint Systems, Inc., Clinical Research, PO Box 92135, Austin, TX 78709, USA

## Abstract

**Background:**

Adhesive Capsulitis (AC) affects patient of all ages, and stretching protocols are commonly prescribed for this condition. Dynamic splinting has been shown effective in contracture reduction from pathologies including Trismus to plantar fasciitis. The purpose of this study was to examine the efficacy of dynamic splinting on patients with AC.

**Methods:**

This controlled, cohort study, was conducted at four physical therapy, sports medicine clinics in Texas and California. Sixty-two patients diagnosed with Stage II Adhesive Capsulitis were grouped by intervention. The intervention categories were as follows: Group I (Control); Group II (Physical Therapy exclusively with standardized protocols); Group III; (Shoulder Dynasplint system exclusively); Group IV (Combined treatment with Shoulder Dynasplint and standardized Physical Therapy). The duration of this study was 90 days for all groups, and the main outcome measures were change in active, external rotation.

**Results:**

Significant difference was found for all treatment groups (p < 0.001) following a one-way ANOVA. The greatest change with the smallest standard deviation was for the combined treatment group IV, (mean change of 29°).

**Conclusion:**

The difference for the combined treatment group was attributed to patients' receiving the best PT combined with structured "home therapy" that contributed an additional 90 hours of end-range stretching. This adjunct should be included in the standard of care for adhesive Capsulitis.

**Trial Registration:**

**Trial Number**: NCT00873158

## Background

Adhesive capsulitis (AC) is an idiopathic disease that affects an estimated 2-6% of the American population (6 to 18 million Americans) [1-6]. It is characterized by fibrosis, decreased volume of the glenoid capsule, pain, and progressive pain with loss of both active and passive Range of Motion (ROM). The direct cost of treating this pathology in the United States in the year 2000 was $7 billion [7] and it affects patients predominantly over 50 years of age.

This condition therefore is a serious pathology, which is also known as "Frozen Shoulder" with three phases: 1) The Painful stage is characterized by the gradual onset of diffuse shoulder pain and which usually lasts one to two months; 2) The Frozen stage is characterized by progressive loss of motion (particularly glenohumeral external rotation) which lasts several months to a year or longer [8]. This stage also exhibits decreased capsular volume which can be visualized with MRI, for differential diagnosis; 3) The Thawing stage is the final stage during which range of motion gradually improves over several months to years. Range of motion deficits may continue to be unresolved for more than 3-5 years following the onset of AC.

Contracture is defined as shortening of connective tissue (ligaments, tendons, and cartilage) caused by excessive arthrofibrosis, immobilization, inactivation, adhesions, or excessive neuromuscular tone [9-11]. Contracture in the shoulder is primarily seen in decreased capsular volume, and is measured with MRI for differential diagnosis. There are many treatment methods for adhesive capsulitis including physical therapy, corticosteroid injections (intra-articular), hydroplasty, manipulation of the joint while under anesthetics and surgery [1-3,5-8,11-20]. The conservative primary treatment for adhesive capsulitis are intra-articular corticosteroid injections and physical therapy, which was examined by Dudkiewicz et al. [8]. They conducted a long-term follow-up (mean 9.2 years) of 54 patients suffering from idiopathic adhesive capsulitis, and their results showed that conservative treatment alone (physical therapy and non-steroidal anti-inflammatory medications) was an effective, long-term treatment method.

Current Treatments for AC range from surgical intervention or manipulation under anesthetics [21], stretching protocols combined with glenohumeral intra-articular corticosteroid injections [22], and continuous passive motion devices [23]. Studies of often report benefits from early intervention [7,12,22] which Earley and Shannon said may help prevent the "downward spiral of forced disuse" leading to contracture [7]. Joint mobilization and flexibility training are common features in treatment of this condition.

Griggs, et al. revealed in a prospective study that showed a significant benefit from participating in a "Four-direction shoulder-stretching exercise program"[12]. In their study, 75 patients diagnosed with Stage II idiopathic adhesive capsulitis participated, and 90% of the patients were satisfied with the outcome of the four-direction shoulder-stretching exercise program. As a long-term study, (ROM measurements were taken at 3, 6, 12,18, and 22 months) ninety percent of the patients gained significant increases in ROM of external rotation, internal rotation, flexion and abduction in the first few months and maintained the ROM through a daily four-direction shoulder-stretching exercise program.

The protocol of using low-load prolonged-duration stretch, combined with the therapeutic principle of increased time at end range allows the patient to reduce contracture by achieving permanent elongation of connective tissue [9,10,13,14,24]. The protocol of increasing total end range time has been shown to be beneficial, despite the cause of contracture in the shoulder joint [7,9,10,25,26]. This is the protocol used with the Dynasplint Systems, (Dynasplint Systems, Inc., Severna Park, MD) and twenty-five years ago a biomechanically correct device was developed to utilize a low-load prolonged-duration stretch with dynamic tension to reduce contracture of the elbow and knee joints [10,24]. This stretching protocol was subsequently included in the Shoulder Dynasplint systems (SDS) modality which allows patients to stretch in flexion, abduction, external, or internal rotation.

**Figure 1:**
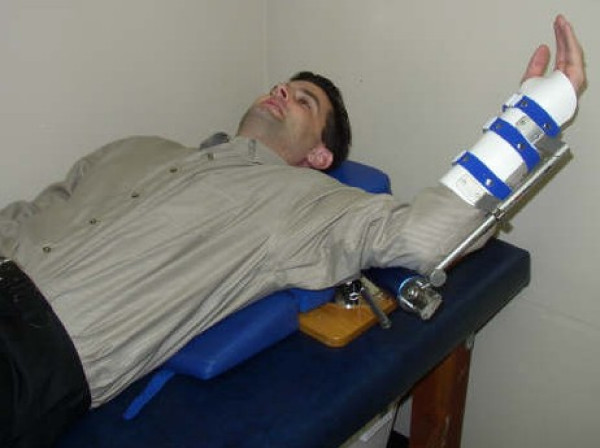
**Dynasplint Shoulder System in External Rotation**.

The SDS is often prescribed as home therapy because it designed to help the patient stretch the shoulder in multiple planes. (See figure 1.) The purpose of this study was to examine the efficacy of dynamic splinting with low-load, prolonged-duration of stretching on adhesive capsulitis. This study used a three-month duration which measured change in the active external rotation of the shoulder, (supine position with humerus abducted to 90°) which is the most common ROM deficit in AC [2,3,5].

## Methods

### Subjects

Sixty-two patients between the ages of 36 and 75 with Stage II adhesive capsulitis were prospectively recruited by referral from clinicians in California and Texas (Mean age 55.6 ± 7.9) and all patients had deficits in external rotation. All patients enrolled had been previously treated with cortical steroid injection(s) but no patients had previously undergone manipulation or surgery. Patients were give informed consent and all patients' rights, protection, and privacy have been ensured in this study as required by the Gaspar-PT Biomed IRB and ethical approval was received from this IRB. Patients were independently prescribed treatment in one of the following groups:

• Group I was the control group, and these patients were only treated with cortical steroid injections, (n = 15).

• Group II patients were treated exclusively with standardized physical therapy, twice per week, (n = 15).

• Group III patients were treated exclusively with the SDS as "home therapy," (n = 16).

• Group IV patients were treated with both physical therapy (twice weekly) and the SDS for daily end-range stretching, (n = 16).

When enrolled, patients were instructed that if they required additional treatment such as additional cortical steroid injections then their participation in this study would be completed but only two patients required such additional treatment methods. Standardized Training and reporting was used for all patients in all groups. All subject data was transmitted in confidential documents without jeopardizing the patients' privacy according to the federal health information privacy protection act.

### Clinical Protocols

All treatment categories were prescribed by the attending physicians rather than being randomized, which may reflect current treatments in use. Physical therapy was standardized, based on the protocols of Vermuelen, Hsu, and Mulligan.[4,6,16,17] These methods included moist heat, patient education and re-evaluation of symptoms, joint mobilization (limited to progressive end-range joint mobilization), passive range of motion, AROM and PNF, and therapeutic exercise. Group II and Group IV patients participated in physical therapy for two or more times per week, and the SDS was worn twice a day, seven days per week.

Group III and Group IV patients who wore the SDS received a standardized treatment protocol and wearing schedule. These patients were instructed on the use by the by the physical therapist and a Dynasplint consultant who accomplished a customized fitting of the unit and taught the standardized protocol regarding how to increase tension in the direction of external rotation, with humeral abduction to 90 degrees. Each subject was instructed to fax a weekly tracking form to investigators which reported daily duration(s) in the SDS and tension settings used.

Patients were instructed to begin the dynamic splinting with only the tension setting of #1 for the first week for accommodation, and then they increase the tension setting to #2 which equals 3.0 foot pounds of force. During this period, patients were instructed to increase the duration in the SDS unit for 20-30 minutes, twice each day (with the goal of stretching 60 total minutes per day).

If the patient had post-wear discomfort or stiffness lasting more than one hour after removing the splint, the duration of the treatment was then reduced for the next two scheduled stretching bouts. After the patient was able to tolerate 60 minutes of stretching, (30 min, bid) then the patient was then instructed to increase the tension every two weeks as tolerated, without discomfort lasting more than one hour following each stretching session.

After 90 consecutive days in the SDS the patients' Active External Rotation was measured again by the same, prescribing clinician. While treatment with the SDS may be performed in multiple planes, this study chose to only evaluate ER rotation because it is the most common restricted ROM from Adhesive Capsulitis [5-7]. Analysis of "Intention to Treat" include the data from patients who were non-compliant (less than 90% PT attendance and/or less than 90% scheduled use of SDS) or did not complete the study duration. All patients' data was included in this analysis.

### Data Analysis

The dependent variable was the change in Active Range of Motion, Supine External Rotation (Humerus abducted to 90°), and the independent variables were groups (Control vs. Physical therapy vs. SDS vs. Combined SDS and Physical Therapy). One-Way Analyses Of Variance (ANOVA) was performed using the Graph-Pad, InStat software, and post-hoc T-tests were performed to measure difference between groups. (An alpha level of 0.05 was used for all tests.) All other calculations were made with the Microsoft Excel program.

### Equipment Used

Dynasplint^® ^Shoulder System

Dynasplint Systems, Inc.

770 Ritchie Highway, Suite W21

Severna Park, MD 21146-4152

800-638-6771

Dynasplint^® ^and Dynasplint Shoulder System

Are registered trademarks of Dynasplint Systems, Inc.

In-Stat Software

2055 Gateway Place, Suite 150

San Jose CA 95110

408-345-4495

## Results

**Figure 2:**
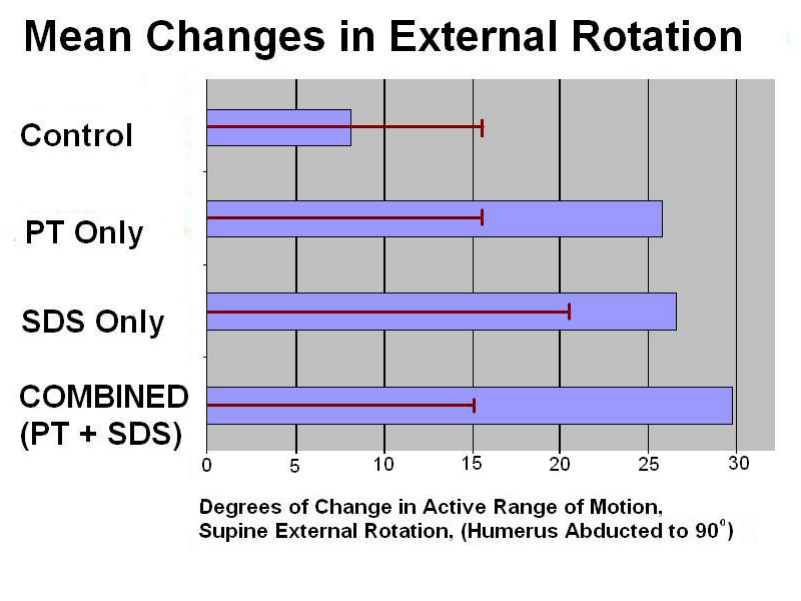
**Graph displaying the results of the study**.

There was a significant difference for each treatment groups (PT Only: T = 4.441, P < 0.001; SDS Only: T = 4.887, P < 0.001; Combined: T = 5.318, P < 0.001). Due to the low power there was not a significant difference between treatment groups but the greatest change and the smallest Standard Deviation was seen for the Combined Treatment Group PT + SDS (mean 29.8° of change and SD = 12.36; see Figure 2.) This suggests that the SDS is a measurably effective adjunct to physical therapy as a structured home therapy. (See Table 1.)

**Table 1:** Active Range of Motion in External Rotation

	**Control**		**PT Only**		**SDS Only**		**Combined**	
	**Initial**	**Final**	**Initial**	**Final**	**Initial**	**Final**	**Initial**	**Final**
	
**Mean**	39.3	47.6	38.3	64.0	38.9	65.3	40.8	70.6
**SD**	15.3	15.6	15.3	15.5	15.3	20.3	15.1	15.1
**N**	15		15		16		16	

## Discussion

The purpose of this study was to examine the efficacy of dynamic splinting on adhesive capsulitis in a prospective, cohort study. Although dynamic splinting for other extremity joints have been studied [10,24], this is the first controlled study investigating the effects of the dynasplint shoulder system. The results showed the efficacy of dynamic splinting as an effective "home therapy" adjunct to physical therapy. The additional 80 to 90 hours of end-range stretching as home therapy combined with standardized physical therapy is considered to be responsible for the greatest change in AROM of external rotation.

The results were in agreement with the study by Griggs et. al. [9], which demonstrated that a conservative treatment protocol of four-direction shoulder-stretching exercise program would benefit shoulder flexibility. This experiment also confirmed the findings of Dudkiewicz et al.[6] which described the efficacy of "conservative protocols." Because ROM deficits frequently exist in external rotation, this experiment chose to examine only that plane following treatment with physical therapy and/or the SDS.

## Conclusion

Use of the SDS may be an effective adjunct "home therapy" for adhesive capsulitis, and the additional 60 minutes per day of low-load, prolonged-stretch was beneficial. (The mean time recorded was 85 hours in this 90 days study.) Earley and Shannon [7] proposed that conservative interventions of adhesive capsulitis would be the most beneficial when initiated as soon as the diagnosis is made, and DS could be an effective initial modality of conservative treatment.

Confounding variables in this study included lack of randomization. Grouping was done by the prescribing clinician who may have been biased, based on patient history. The total duration that each patient endured Stage II of adhesive capsulitis was not differentiated. Limitations of this study also included that it was only performed on a small number of patients and was limited to examining the Active Range of Motion, Supine External Rotation (Humerus abducted to 90°).

A future study comparing the duration of treatment to discharge between groups would be greatly beneficial in measuring the benefits that the SDS has in treating adhesive capsulitis. A larger subject population would reduce the chance for type two error, and a randomized, controlled trial would eliminate most of the limitations discussed regarding this study. A pain scale should also be used in the next study.

## List of Abbreviations

(AC): Adhesive capsulitis; (ROM): Range of Motion; (SDS): Shoulder Dynasplint systems; (ER): External Rotation; (ANOVA): Analyses Of Variance; (PT): Physical Therapy.

## Competing interests

PDG has no competing interest and has received no financial compensation for this study or manuscript. FBW is employed by the parent company of Dynasplint systems but he has no stock or vested interest in either company.

## Authors' contributions

PDG was responsible for experimental design, patient recruitment and treatment, and development of this manuscript. FBW contributed in experimental design, study coordination, data analysis, and development of this manuscript. Both authors read and approved the final manuscript.

## Pre-publication history

The pre-publication history for this paper can be accessed here:


